# Undoing Self‐Contempt in Borderline Personality Disorder: A Controlled Test of Change in Emotion Processing in Psychotherapy

**DOI:** 10.1002/cpp.70335

**Published:** 2026-09-18

**Authors:** Ueli Kramer, Loris Grandjean, Hélène Beuchat, Stéphane Kolly

**Affiliations:** ^1^ University Institute of Psychotherapy, Department of Psychiatry Lausanne University Clinic and University of Lausanne Lausanne Switzerland; ^2^ Department of Psychology University of Windsor Windsor Canada; ^3^ General Psychiatry Service, Department of Psychiatry Lausanne University Clinic and University of Lausanne Lausanne Switzerland

**Keywords:** assertive anger, borderline personality disorder, brief treatment, emotion processing, good psychiatric management, mechanism of change, self‐compassion, self‐contempt

## Abstract

**Background:**

Emotion processing has been discussed as a mechanism of change in several forms of psychotherapy for a broad range of psychological disorders. The general conclusions of the field do not allow for a step‐by‐step granularity of relevant emotional experiences in clients. This is particularly important for personality disorders, especially borderline personality disorder (BPD). Using a controlled out‐of‐therapy assessment, we assume that the lessening of expressed self‐contempt by clients gives way to proportionally more expressions of self‐compassion and assertive anger after a brief psychiatric intervention.

**Methods:**

The present secondary analysis of a randomized controlled trial includes *N* = 79 participants (*n* = 27 clients with BPD in the active treatment following the principles of good psychiatric management [GPM]; *n* = 23 clients with BPD in the treatment as usual [TAU]; *n* = 29 healthy controls). Both treatments—GPM and TAU—lasted 4 months. All participants underwent out‐of‐therapy experiential two‐chair dialogues focusing on self‐criticism at two time points. The participants' emotional expressions were assessed using the video‐rated Classification of Affective Meaning States and the Self‐Contempt Rating Scale.

**Results:**

The results indicated that the intensity of out‐of‐session observed self‐contempt as part of a person‐relevant self‐criticism decreased in clients with BPD undergoing GPM, with a large effect, similar to healthy controls. A medium‐sized effect was found for reduction in self‐contempt in clients undergoing TAU. The results also indicated that the proportion of individuals with BPD undergoing GPM accessing self‐compassion and assertive anger increased, which was not the case for healthy controls nor clients in TAU.

**Conclusions:**

Undoing self‐contempt may be an elusive, yet central task in psychotherapy for clients with BPD. The present study explored the client process of change of undoing self‐contempt and moving towards self‐compassion and assertive anger. These conclusions bear important clinical and research implications.

## Summary

- Self‐contempt in borderline personality disorder may have to be assessed early in therapy.
- Addressing and reducing self‐contempt may be a central clinical task to promote healthy emotional processing.
- Fostering primary adaptive emotional responses in borderline personality disorder may be an important therapeutic task in psychotherapy.

## 1 Background

Emotion is key to understanding how psychotherapy works. So far, three meta‐analyses conclude that clients' in‐session emotion processing positively impacts psychotherapy outcomes across types of treatment, type of theory, assessment tool and type of psychological problem treated (Daros et al. 2021; Peluso and Freund 2018; Sonderland et al. 2023). Peluso and Freund (2018) focused on both the clients' emotion processing and the therapist's in‐session fostering of emotional changes. A fourth meta‐analysis focused on the direct effect of therapist working with emotion in psychotherapy, with demonstrated effects across therapy approaches (Iwakabe et al. 2024). Taken together, it suggests that emotion processing is a key candidate mechanism of change explaining outcome in psychotherapy (Doss 2004; Kazdin 2009; Kramer, Levy, and McMain 2024; Samson et al. 2024). Conclusions from some of this work have been taken up by a workgroup of the American Psychological Association, suggesting that fostering emotion processing, as well as regulation, in psychotherapy was recommended from a transdiagnostic perspective (Teachman and Muran 2024).

### 1.1 Sequences in Emotional Experiences in Psychotherapy

Among the different functions central to emotion change in psychotherapy, sequences of emotion changes are particularly important (Greenberg 2019; Greenberg and Pascual‐Leone 2024; Pascual‐Leone and Greenberg 2007; Pascual‐Leone 2018, 2026). These sequences denote the step‐by‐step changes in the client's emotional experiences, from less adaptive (i.e., secondary and instrumental) emotions to primary adaptive emotions. Psychotherapy process analyses yielded a model of nine steps, where the client moves from global distress (i.e., undifferentiated emotion with low meaning; a less adaptive emotion), over fear or shame (i.e., specific self‐related emotions) and the access of the client's need (i.e., awareness of the goal, concern or relevant content to the individual) to the adaptive affective‐meaning units (i.e., assertive anger aiming at expressing healthy anger to edify boundaries related to one's identity, or grief aiming at letting go of a specific, highly relevant to the individual, content or person). Pascual‐Leone (2009) showed that clients who resolve their emotional difficulty do not evolve through this sequence in a linear fashion, but in a two‐step‐forward‐one‐step‐back fashion. Clients who were able to access more healthy and adaptive emotional steps were those who benefitted more from treatment across diagnoses (Pascual‐Leone 2018). For the reduction in self‐criticism using chairwork, Delatraba et al. (2025) demonstrated that the named emotion sequence (i.e., specifically adaptive affective meaning‐units following fear or shame) predicted psychotherapy outcome and mediated the link between therapist focus on emotion and outcome.

### 1.2 Emotion Processing in Psychotherapy for Borderline Personality Disorder (BPD)

Psychotherapy is the first‐line intervention for BPD (Setkowski et al. 2023; Storebø et al. 2020), with comparable effects across treatment models, for example, dialectical‐behaviour therapy (DBT), transference‐focused psychotherapy (TFP) and also generalist treatments (such as GPM; see also for a review Kramer 2025). All therapy models focus, at least to some extent, on supporting better emotion processing in clients through a variety of therapist interventions.

Emotion processing is particularly relevant in psychotherapies for clients with BPD, as it is assumed that its occurrence may be explained by, among other factors, emotion dysregulation in the context of attachment relationships (Gunderson et al. 2018; Rudge et al. 2020; Pos et al. 2012). BPD is marked by intense, negative self‐judgements and very harsh self‐criticism, which contribute to a fragile self‐concept, unclear self‐identity and problematic interpersonal transactions (Pos et al. 2012). Interpersonal hypersensitivity represents a hypothesized core feature of clients with BPD (Gunderson and Lyons‐Ruth 2008). This model proposes that distinct attachment‐based self‐states organize the client's emotional experiences, explaining their problematic interpersonal responses, extreme fragility in self‐identity and self‐esteem, along with self‐harming behaviour.

Several studies showed that in‐session emotion sequences explained the outcome across psychotherapies for BPD. Focusing on DBT in the context of a randomized controlled trial (RCT), Kramer, Pascual‐Leone, Berthoud, et al. (2016) compared two types of anger with regard to their potential to mediate change in psychotherapy, according to the definitions by Pascual‐Leone and Greenberg (2007) and Pascual‐Leone (2018): assertive (healthy adaptive) and rejective (less healthy secondary) anger. The results showed that the frequency of in‐session healthy anger increased in the DBT condition (whereas no change was found in individuals in the treatment as usual [TAU] condition). Assertive anger mediated the decrease in problems in the social, family and professional domains. In a recent and larger process‐outcome study based on an RCT, Nardone et al. (2024) focused on the entire emotion sequence (according to Pascual‐Leone 2018, explained above) in DBT for BPD and found the in‐session frequency of self‐compassion or self‐soothing (i.e., an adaptive form of an affective meaning state) to be predictive of symptom decrease. The emotion sequence was also studied in good psychiatric management (GPM) for BPD. Using data from an RCT on the role of case formulation on treatment outcome, Berthoud et al. (2017) showed that from the initial therapy session on, changes in emotion categories—moving from global distress to any other, more differentiated and nuanced, emotion expression—were systematic. These predicted a reduction in interpersonal problems, in the condition where the therapist used a specific case formulation. GPM puts the notion of interpersonal hypersensitivity at its core and proposes psychoeducation intervention to clients with BPD to raise awareness about links between their experienced and expressed emotions and specific attachment states, as they self‐organize in their daily lives related to interpersonal triggers, so these changes were expected by the treatment model.

### 1.3 Self‐Contempt as a Particularly Harmful Emotional Experience in BPD

Fragile self‐processes in BPD are often marked by the expression of self‐contempt, yet this specific emotion is oftentimes overlooked (Aguirre 2025; Winter et al. 2017; Wilner et al. 2024). Self‐contempt may be defined as cold self‐hatred and fundamental disrespect of oneself, which some individuals may express in a subliminal fashion when facing situations of defeat, low self‐image and failure (Miceli and Castelfranchi 2018). Self‐contempt may be a particularly destructive emotion state when it appears in the therapeutic process (Whelton et al. 2007). It is associated with severe symptoms of BPD, such as self‐harming behaviour (Uh et al. 2021) and suicidal thoughts and behaviours (Aguirre 2025; Rüsch et al. 2019). Sallin et al. (2021) used criteria to assess expressed self‐contempt in‐session (Kramer and Pascual‐Leone 2016; Whelton and Greenberg 2005). This study wanted to know if in‐session self‐contempt changes across a brief psychotherapy for BPD (i.e., GPM). The study did not find any change over the course of brief treatment, but Sallin et al. (2021) showed that the intensity of expressed self‐contempt was related to the intensity of borderline symptoms. Although self‐contempt was not directly associated with outcome, it did predict the evolution of the therapeutic alliance, differentially rated by the client and the therapist. For the client, as predicted, the intensity of self‐contempt was negatively associated (see Whelton et al. 2007); for the therapist, surprisingly, the intensity of self‐contempt was positively associated. The researchers speculated that the therapist's response to harsh self‐contempt may evoke a particularly strong affective therapist engagement and empathy. Arguably, expressed self‐contempt is related to the client experiencing shame (as a core maladaptive emotion in the sequence proposed by Pascual‐Leone 2018) in response to the self‐critical content. For social anxiety, Haberman et al. (2019) studied the undoing of the client's expressed shame in a 27‐session‐long emotion‐focused therapy (EFT). This study found that, although the average number of minutes spent in shame (per therapy hour) decreased over therapy, the average number of minutes spent in assertive anger increased. Undoing shame by assertive anger may represent an in‐session process resolving social anxiety, but the role of self‐contempt in the process of the criticism itself has not been elucidated in this study. It appears from these lines of research that self‐contempt may be a mostly hidden, but central, emotion state, in particular for clients with BPD and other fragile self‐processes (Aguirre 2025; Sallin et al. 2020; Wilner et al. 2024).

### 1.4 The Need to Study Out‐Of‐Session Emotion Processing

The studies discussed above assessed the in‐session therapeutic processes, but not the out‐of‐session mechanisms that could explain the outcomes and lasting changes. This is a crucial distinction, as Doss (2004) defines a mechanism of change as the interplay between the processes observed in‐session with the client's generalized skill level *outside of the therapy hour*, which is the variable that mediates the clinical change. Also, assessing emotion in‐session is affected by the therapist's responsiveness and intervention (i.e., a therapist focusing on emotion may evoke certain emotions more than a therapist who does not work in this way; Pascual‐Leone and Kramer 2023). Kramer, Grandjean, et al. (2024) addressed this problem by conducting, within an RCT, emotion‐evoking two‐chair dialogues outside of the therapy hours (Whelton and Greenberg 2005; Kramer and Timulak 2024) with all participants over the course of treatment within a controlled paradigm focusing on self‐criticism. They found that advancement on emotion sequences (according to Pascual‐Leone 2009) mediated the effect of a psychiatric intervention at the level of the reduction of interpersonal problems (Kramer, Grandjean, et al. 2024). This effect of the advancement on emotion sequences was found for both treatments in the study (brief GPM and TAU), but the effects for the active treatment, or GPM, were larger, and the mediation effect was specific to GPM. This means that GPM functions based on its focus on the link between clients' interpersonal hypersensitivity and emotions; it also means that TAU may have an overlooked effect in psychotherapy trials. Specifically for GPM, Grandjean, Rohrbach, et al. (2025) showed that this active treatment was associated with an increasingly larger variance in out‐of‐session emotion arousal when the client was invited to criticize oneself, when compared to TAU. Large variance in arousal was associated with better mental health indices. Grandjean, Beuchat, et al. (2025) pushed this line of research to the level of the *out‐of‐session* brain correlates of the emotion responding to self‐criticism. They found that the neurofunctional activations related to the self‐critical words used by the individuals corresponded to the activations the individuals had when facing generic negative stimuli, namely, at the level of an activation in the left anterior insula, a structure associated with the representation of the body aspects of the emotional experience. In all these studies, GPM was studied as a specific treatment for reducing the problems associated with BPD and at the same time providing a scalable and largely disseminable clinical framework (Choi‐Kain et al. 2016; Gunderson 2016; Gunderson and Links 2014; Iliakis et al. 2019). It remains an open question what specific pathway of change *out‐of‐session* expressed self‐contempt takes in these brief psychiatric treatments, as compared to treatments without the deliberate in‐session focus on the client's interpersonal hypersensitivity (and on the emotional experience), as compared to normative (healthy) controls over time. We assume that the emergence of assertive anger and self‐compassion (in the out‐of‐session assessment in response to the self‐criticism) are essential components reserved for the deliberate therapeutic work on interpersonal hypersensitivity and emotion.

### 1.5 The Present Study

Given this body of research, it is necessary to study change in the frequency of selected emotion categories of the course of psychotherapy for BPD, in a controlled *out‐of‐session* paradigm. We assumed that (H1) over the course of a brief GPM for BPD, the intensity of expressed self‐contempt—measured in a context while the client criticizes themselves—decreased over the course of treatment. We assumed that the (H2) expressed self‐compassion and the (H3) assertive anger—measured out‐of‐session in response to the expressed self‐criticism—increased over the course of treatment. These changes should be specific to the active psychiatric treatment (GPM), as compared to a (less emotion‐ and interpersonally evoking) TAU and compared to normative healthy controls. Fluctuations in state self‐esteem are expected within each two‐chair dialogue task (without hypothesis, as a control of the assessment procedure).

## 2 Methods

### 2.1 Context

The present secondary analysis of an RCT drew from the client sample with BPD who were randomized either to a 4‐month brief GPM or to a 4‐month brief TAU (Kramer, Grandjean, et al. 2020; Kramer, Grandjean, et al. 2024). The study was conducted in a public psychiatry department. Inclusion criterion for the parent study was a diagnosis of BPD according to the Diagnostic and Statistical Manual of Mental Disorders, Fifth Edition (APA 2013); exclusion criteria were neurocognitive disorder, psychotic spectrum disorders and bipolar disorder I. Clients were diagnosed using SCID‐5‐PD (APA 2013; with good inter‐rater reliability). The current study was approved by the ethics committee CER‐VD (2017–02167). Informed consent was given by all participants in writing to publish the results of the present study.

### 2.2 Clients, Therapists and Raters in the Present Study

A total of *N* = 50 clients with BPD were included in the current study. From the initial sample of the parent study, they were selected based on the criteria that they needed to have at least two complete experiential two‐chair dialogues as part of the out‐of‐therapy assessment procedure (*n* = 26 clients with only one such assessment point were excluded from the present study). The current clinical sample comprised *n* = 27 clients who underwent GPM and *n* = 23 who underwent TAU. In addition, a total of *n* = 29 age‐matched healthy controls were recruited. Exclusion criteria for the latter were any currently known psychopathology or any current psychotherapeutic treatment. They underwent the same assessment procedures as the clients during the same time frame.

The clinical groups did not differ in terms of age (GPM: 32.85 years [9.65; range between 19 and 53]; TAU: 30.92 years [7.20; range between 23 and 50]), nor in terms of sex (GPM: 21 women [78%]; TAU: 15 women [65%]). The comorbidity profiles corresponded proportionally to the distribution in the main study (GPM: 62% depression, 7% panic disorder, 4% anxiety disorders, 15% SUDs, 26% eating disorders and 15% any other personality disorder; TAU: 61% depression, 13% panic disorder, 9% anxiety disorders, 30% SUDs, 61% eating disorders and 22% any other personality disorder).

A total of *N* = 18 therapists treated the clients. The therapists received at least 1 day of clinical workshop on applying GPM principles; supervision was offered weekly by certified GPM trainers.

A total of *N* = 3 trained raters contributed. Two were Master's‐level students, and one was a lead psychologist (PhD). They received research training in the rating scales used for the present study.

### 2.3 Interventions

Brief good or general psychiatric treatment (GPM; Gunderson and Links 2014; Charbon et al. 2019) is a low‐resource intervention specific to BPD. It involves straightforward discussion about the BPD diagnosis and comorbidities, and the use of the interpersonal hypersensitivity model, which enables making links between interpersonal triggers, emotions, thoughts and actions. Four attachment states (secure, threatened, isolated and despairing) are related to BPD symptoms and the client's emotions in specific situations (fear, shame, contempt, anger, sadness, for example). The last session is a synthesis of the treatment and a decision for next steps of treatment, if needed. Some clients required more treatment and were referred to evidence‐based psychotherapy or additional GPM.

TAU consisted of crisis intervention as usual, safety management, in accordance with the minimal ethical standards and the guidelines of good medical practice. It was proscribed to discuss the BPD diagnosis and the client's interpersonal hypersensitivity model, nor to focus on emotion explicitly. Therefore, in situations in which the attachment states were apparent, therapists were invited to acknowledge the difficulty, without linking them to the BPD diagnosis nor to the client's emotions. Both treatments were delivered once weekly, individually, across 4 months.

Adherence to GPM principles in the GPM condition (and non‐adherence to GPM principles in the TAU) was assessed using a specific self‐report questionnaire. The results were satisfying, consistent with prediction, and differentiated significantly between the two conditions in the present sample (Kramer, Grandjean, et al. 2024).

### 2.4 Assessments

#### 2.4.1 Paradigm and Observer‐Rated Assessments

Participants in the present study underwent an out‐of‐session assessment enabling the observation of expressed emotion in a personally relevant context. The therapeutic task from EFT, focusing on the elaboration of one's self‐critical processes, was used (Greenberg 2015). Assessors were trained in this task and were supervised by a recognized isEFT (international society for EFT) supervisor. Two steps compose this emotion‐evoking experiential task (Kramer and Pascual‐Leone 2016; Whelton and Greenberg 2005): (1) the client is invited to experience—in imagination—as vividly as possible a situation of failure from their past and (2) the investigator conducts a two‐chair dialogue focusing on the self‐critical voice related to the specific situation. This task was conducted repeatedly (at intake and at 2 months into the treatment for the present study) and videotaped at each instance. We selected the two time points close to the beginning of the treatment, as (a) we are interested in the earliest possible out‐of‐session changes, and (b) the effects reported by Kramer, Grandjean, et al. (2024) were found between Months 0 and 2. The State‐Self‐Esteem Scale (SSES; see below) was used to control for the effectiveness of the laboratory manipulation. For each two‐chair dialogue assessment, participants filled in the SSES three times: (a) before the imagination task, (b) right after the imagination task and (c) after the two‐chair dialogue focusing on self‐criticism. We assume that the state self‐esteem is lowest at b, compared with a and c. The two‐chair dialogues were supervised, using video footage, in order to ensure the comparability of the instructions across participants. Investigators of the two‐chair dialogues were unaware of the study hypotheses, as the latter were only developed once the trial was complete.

The *Classification of Affective Meaning States* (CAMS; Pascual‐Leone and Greenberg 2005) is an observer‐rated scale assessing distinct emotion types in the form of affective‐meaning states. These states can be ordered into a sequence (Pascual‐Leone 2009). For the present study, among the nine distinct emotion categories, we selected two: (a) Self‐Compassion and (b) Assertive Anger. They are both part of the advanced meaning‐making components and represent essentially primary adaptive emotions (Greenberg 2015). Each of the two emotion categories was coded in the present using a present‐absent (1–0) assessment for each session. This was selected based on eight pilot cases from an earlier study with consistent procedures (Kramer et al. 2018). These assessments showed that the actual two‐chair dialogue as assessment (between assessments b and c above) was relatively brief, varying between 10 and 20 min. Although this is in keeping with clinical practice in EFT, each emotion state clients are in typically emerged while responding to their self‐criticism, *once* during 2 min (according to the rating criteria of the CAMS). Based on this empirical observation, we decided that the most relevant variable in the present study was the presence/absence of Self‐Compassion and Assertive Anger for each assessment.

The Self‐Contempt Rating Scale (SCRC; Beuchat et al. 2024) is an observer‐rated scale assessing the intensity of the self‐contemptuous process in a session. It uses a 5‐point Likert scale with a specific manual for coding. Rating criteria are grounded in emotion research (Ekman and Friesen 2003) and observational process research in psychotherapy (Whelton and Greenberg 2005; Kramer and Pascual‐Leone 2016; Sallin et al. 2021). The rating criteria involve markers on the (a) verbal, (b) para‐verbal and (c) non‐verbal levels. The coding is possible in variable time units. Earlier research has shown sufficient internal validity of the scale, as well as good inter‐rater reliability for clients and healthy controls (Beuchat et al. 2024). The intensity of self‐contempt is assessed here using one score (ranging between 1 = absent and 5 = very intense) per session.

A total of *N* = 3160 min of two‐chair dialogues across the two time points were analysed. A total of 75% of the two‐chair dialogues were analysed by pairs of two raters for each score (out of the three raters), yielding, for the CAMS, an average inter‐rater reliability of intra‐class coefficient (1, 2) = 0.78, and for self‐contempt a kappa = 0.79.

#### 2.4.2 Self‐Report Questionnaire

State Self‐Esteem Scale (SSES; Heatherton and Polivy 1991) is a self‐report questionnaire using 20 items to assess moment‐by‐moment changes in self‐esteem, using a 5‐point Likert scale. Validity of the scale, as well as its sensitivity to laboratory manipulations, and high internal consistency (Cronbach alpha = 0.92) were shown by Heatherton and Polivy (1991). In the present study, we used the overall mean score for each time point. Cronbach's alpha for the current sample was 0.85.

### 2.5 Procedures

After the outcome trial was completed, the video material of the two‐chair dialogues was compiled and rated. Statistical analyses for H1 involved a series of paired‐sample *t*‐tests (pre‐post assessments of the intensity of self‐contempt), in addition to between‐condition *t*‐tests comparing clients with healthy controls and comparing the clients in GPM with the clients in TAU. For H2 (proportions), we conducted a series of paired‐sample proportion McNemar tests (pre‐post assessments of the proportion of presence of self‐compassion), in addition to a series of chi‐square tests comparing clients with healthy controls and comparing the clients in GPM with the clients in TAU. For H3 (also proportions), we conducted a series of paired‐sample proportion McNemar tests (pre‐post assessments of the proportion of presence of assertive anger), in addition to a series of chi‐square tests comparing clients with healthy controls and comparing the clients in GPM with the clients in TAU. If the numbers per cell in the chi‐square comparisons were under 5, it is recommended to use Monte Carlo simulated values to verify the results. This was done and yielded comparable results (we only report the original results in this case). The data structure was nested (clients nested within therapists, and partially within coders). In order to account for this situation, the parent study conducted multi‐level analyses on the emotion processing variables and did not find statistically significant coefficients for the hierarchical structure. Therefore, we decided to model these components in the present study. Statistical analyses were carried out using SPSS Version 29 (IBM 2023).

## 3 Results

Preliminary statistical analyses involved testing the impact of missing values on the emotion processing variables and testing between‐condition and between‐group effects on the state self‐esteem.

The presence of missing values of the emotion processing variable, when comparing client completers with non‐completers from the original sample (*N* = 76; Kramer, Grandjean, et al. 2024) did not yield any significant differences between the two sub‐samples at both time points studied in the present research (at intake: *F* (1, 76) = 1.67, *p* = 0.20; at 2 months: *F* (1, 50) = 0.47, *p* = 0.50). The focus on those with at least two time points completed (*N* = 50 clients in the two conditions) is therefore justified.

Clients from the clinical sample reported more problems in self‐esteem than healthy controls at both time points (for the first time point, *t* = 4.19, *p* = 0.00+). For the comparison between clients in GPM vs. TAU, between‐condition differences were present at the pre‐treatment two‐chair dialogue, with more self‐esteem problems reported in TAU than in GPM (e.g., *t* = 2.34, *p* = 0.02). This difference was not significant at the 2‐month two‐chair dialogue (e.g., *t* = 0.79, *p* = 0.44). Separate paired‐sample *t*‐tests for clients in GPM and clients in TAU showed a consistent pattern of within‐person changes: Systematically, significantly more self‐esteem problems are reported at assessment moment (b) (right after the imagination task), for both groups and at both pre‐treatment and at 2 months. Separate paired‐sample *t*‐tests for healthy controls showed that no such effect was found in healthy controls (no within‐person differences within each task at pre and post, with stable and high self‐esteem scores). We can conclude from these preliminary analyses that the laboratory manipulation of the imagination using the two‐chair dialogue on self‐criticism had the anticipated effect on state self‐esteem and was successful for the clients. For the healthy controls, high and stable scores on state self‐esteem were found.

For the first hypothesis, the first paired‐sample *t*‐test revealed that the intensity of self‐contempt decreased over the course of the treatment, with a large effect size (*d* = 0.84). A comparable reduction over time, without treatment, albeit on a significantly lower level of intensity, was observed in the healthy controls over the same time window (*d* = 0.81; see Table 1A). The second paired‐sample *t*‐test revealed that the intensity of self‐contempt decreased in clients undergoing GPM, with a large effect size (*d* = 0.91). A much smaller reduction in the intensity of self‐contempt, and also at a significantly lower level of intensity, was found in the TAU, with a medium effect size (*d* = 0.50; see Table 1B).

**TABLE 1A:** Self‐contempt for clients and in healthy controls (*N* = 79).

	Clients (*n* = 50) Mean (SD)	Controls (*n* = 29) Mean (SD)	*t*‐test	*p*
Time 1	4.52 (0.67)	4.00 (0.83)	5.26	0.00+
Time 2	3.62 (0.82)	3.31 (0.60)	3.90	0.00+

*Note:* Paired‐sample *t*‐test for clients: *t*(49) = 4.38, *p* = 0.00+, *d* = 0.84; paired‐sample *t*‐test for controls: *t*(28) = 2.07, *p* = 0.02, *d* = 0.81.

**TABLE 1B:** Self‐contempt for clients undergoing GPM and TAU (*N* = 50).

	GPM (*n* = 27) Mean (SD)	TAU (*n* = 23) Mean (SD)	*t*‐test	*p*
Time 1	4.67 (0.55)	4.35 (0.78)	1.65	0.054
Time 2	3.81 (0.92)	4.22 (0.67)	1.74	0.04

*Note:* Paired‐sample *t*‐test for GPM: *t*(26) = 4.88, *p* = 0.00+, *d* = 0.91 (test of H1); paired‐sample *t*‐test for TAU: *t*(22) = 1.14, *p* = 0.13, *d* = 0.51.

For the second hypothesis, the first paired‐sample McNemar test revealed that the proportion of clients with self‐compassion as emotion response to self‐criticism increased over the course of the treatment (significant effect at the level of alpha = 0.01). A comparable increase in this proportion over time, without treatment and with comparable proportions, was observed in the healthy controls over the same time window (see Table 2A). The second paired‐sample McNemar test revealed that the proportion of individuals with self‐compassion as an emotion response to self‐criticism increased in clients undergoing GPM (significant effect at the level of alpha = 0.001). An increase in the proportion of individuals with self‐compassion as emotion response to self‐criticism was observed in clients undergoing TAU (significant effect at the level of alpha = 0.001; see Table 2B).

**TABLE 2A:** Presence of self‐compassion for clients and in healthy controls (*N* = 79).

	Clients (*n* = 50) Proportion	Controls (*n* = 29) Proportion	Chi‐square	*p*
Time 1	5	4	0.26	0.43
Time 2	14	13	2.31	0.10

*Note:* Paired‐sample proportion McNemar test for clients: *Z*(50) = 2.32, *p* = 0.01; paired‐sample proportion McNemar test for controls: *Z*(29) = 2.71, *p* = 0.00+.

**TABLE 2B:** Presence of self‐compassion for clients undergoing GPM and TAU (*N* = 50).

	GPM (*n* = 27) Proportion	TAU (*n* = 23) Proportion	Chi‐square	*p*
Time 1	2	3	0.51	0.42
Time 2	14	0	16.56	0.00+

*Note:* Paired‐sample proportion McNemar test for GPM: *Z*(27) = 3.46, *p* = 0.00+ (test of H2); paired‐sample proportion McNemar test for TAU: *Z*(23) = 4.47, *p* = 0.00+.

For the second part of the second hypothesis, the first paired‐sample McNemar test revealed that the proportion of clients with assertive anger as an emotional response to self‐criticism increased over the course of the treatment (significant effect at the level of alpha = 0.001). No increase in this proportion over time, without treatment and with partially lower proportions, was observed in the healthy controls over the same time window (see Table 3A). The second paired‐sample McNemar test revealed that the proportion of individuals with assertive anger as an emotional response to self‐criticism increased in clients undergoing GPM (significant effect at the level of alpha = 0.001). No such increase was found in the proportion of individuals with assertive anger as an emotional response to self‐criticism was observed in clients undergoing TAU (see Table 3B).

**TABLE 3A:** Presence of assertive anger for clients and in healthy controls (*N* = 79).

	Clients (*n* = 50) Proportion	Controls (*n* = 29) Proportion	Chi‐square	*p*
Time 1	7	7	0.26	0.20
Time 2	21	7	2.56	0.09

*Note:* Paired‐sample proportion McNemar test for clients: *Z*(50) = 2.99, *p* = 0.00+; paired‐sample proportion McNemar test for controls: *Z*(29) = 0.00, *p* = 0.50.

**TABLE 3B:** Presence of assertive anger for clients undergoing GPM and TAU (*N* = 50).

	GPM (*n* = 27) Proportion	TAU (*n* = 23) Proportion	Chi‐square	*p*
Time 1	3	4	0.52	0.41
Time 2	19	2	19.39	0.00+

*Note:* Paired‐sample proportion McNemar test for GPM: *Z*(27) = 3.77, *p* = 0.00+ (test of H2); paired‐sample proportion McNemar test for TAU: *Z*(23) = 1.00, *p* = 0.16.

## 4 Discussion

The goal of the present study was to test a particular sequence of emotion processing in brief treatment for BPD, that is, the undoing of self‐contempt, potentially by assertive anger and self‐compassion. We assumed that, for the active treatment using an intervention focused on interpersonal hypersensitivity (GPM) and the client's emotion, when clients underwent the out‐of‐session controlled assessment using the two‐chair dialogue, the intensity of their expressed self‐contempt would lessen. In parallel, we assumed that their emotional response to the self‐criticism outside of the therapy hour would be increasingly marked by self‐compassion and assertive anger, between early and 2 months into treatment. We assumed that these effects were specific to clients with BPD undergoing GPM as the treatment focusing on linking the interpersonal hypersensitivity to the client's emotions.

Hypotheses were partially supported. First, we found that the two‐chair dialogue focusing on self‐criticism is a reliable affect‐evoking procedure that is well suited to modulate self‐states and in particular emotions associated with self‐esteem and identity (such as self‐contempt). The manipulation checks were successful, in particular for the within‐person fluctuations between assessments a, b and c (with most self‐esteem problems at b, right after the imagination task). This was true for the clients, but not for the controls, who maintained a stable and solidly moderate to high level of self‐esteem throughout the task.

### 4.1 Healthy Controls May Undo High Self‐Contempt With Self‐Compassion, but Not Assertive Anger

We examined changes in performance markers over time across all three groups. A consistent process of reducing self‐contempt emerged in all pre‐ to post‐assessment comparisons. However, the specific impact of repeated exposure to the two‐chair dialogue focusing on self‐criticism on emotional performance remains unclear. For clients with BPD *and* for healthy controls, the change between pre and post was large. This may indicate a generic learning effect—a habituation to the specific assessment context: We can assume that the first assessment may be overestimating the real intensity of self‐contempt, which may emerge only after one or 2 months into treatment (at our second assessment point). This seems plausible, in particular for healthy controls who had an average intensity of 4 (out of 5; indicating a moderately intense level of self‐contempt), whereas at the second visit, this score dropped to close to 3.3 (indicating a medium level of self‐contempt). The entire pattern of results observed in the healthy controls is interesting: The self‐esteem is not affected by the two‐chair dialogue (unlike the more vulnerable clients, who had a significant drop in self‐esteem), whereas controls present with rather high performance indicators of self‐contempt. Although the reduction to lower levels of self‐contempt may be explained by a regression to the mean in all three groups, it may have a different impact on emotion processing and self‐esteem in healthy controls than for the clients. The emergence of other types of emotions, more adaptive ones, in parallel (i.e., assertive anger) does not seem to be needed (the state self‐esteem remains unaffected by the process of criticizing oneself), unlike for the clients. This may indicate that healthy controls have a resourceful propensity to defend their identity and self‐esteem in the face of contemptuous stimuli, and the actual expression of related adaptive emotions may not be needed. Although this effect holds true for assertive anger in healthy controls, it is different for self‐compassion. The latter increased substantially between the two time points, much like the clients'. We hypothesized that self‐compassion seems to ‘drive’ most of the undoing of the self‐contempt in healthy controls, whereas for clients, in addition, the emergence of assertive anger may be needed to undo self‐contempt (see below).

### 4.2 Undoing of Self‐Contempt in GPM

Specifically, the study found that clients with BPD undergoing GPM had a large decrease in self‐contempt over the treatment, whereas the clients with BPD undergoing TAU had a medium effect size and remained at a more severe level of intensity of expressed self‐contempt. Working with the client's interpersonal hypersensitivity and specific attachment states, in conjunction with the emotion responses in these concrete situations—as done in GPM—may explain this specific and large effect of reduction in self‐contempt in the clients. These results support the use of an emotional performance task to assess change in such an elusive emotion state as contemptuousness: Sallin et al. (2020) tested change in self‐contempt in psychodynamic therapy sessions and did not find any progression over the course of treatment. This may be because of the interaction between emotion processing and the therapeutic alliance: Expressing such destructive emotion states may both require a strong therapeutic alliance and may also put it at risk—in the eye of the client and under certain conditions. As such, some clients may inhibit the expression of these states in a dyadic therapeutic relationship, which may result in an inconsistency of the assessments: An out‐of‐session performance task, such as used in the present study, may address these issues.

An interesting point here is the emotion of (maladaptive) shame, potentially underlying the self‐critical dialogue (Haberman et al. 2019; Kramer and Pascual‐Leone 2016), in particular when self‐contempt is expressed. Although the present study did not include (maladaptive) shame in the sequence studied, we cannot rule out that inner shame (expressed or not in the response to self‐contempt) may play a crucial role in determining the process of undoing self‐contempt. This would involve a three‐step sequence, with self‐contempt as a starting point (accompanying the self‐criticism directly), followed by the access to maladaptive shame and then to the adaptive emotions (e.g., self‐compassion or assertive anger). As shown in other studies (Haberman et al. 2019; Kramer, Pascual‐Leone, Rohde, and Sachse 2016), maladaptive shame may need longer psychotherapy processes (i.e., 20 to 40 sessions) to emerge as a relevant experience.

### 4.3 Emergence of Self‐Compassion and Assertive Anger in Clients With BPD

Consistent with the predictions from the sequential perspective on emotion processing (Pascual‐Leone 2009)—which indicates that in psychotherapy, although one emotion category lessens, others emerge and take centre stage dynamically over time—the current study focused on the emergence of self‐compassion and assertive anger as supporting the undoing of the intensity of self‐contempt in the two‐chair dialogue.

The emergence of clients' self‐compassion in the current study is consistent with the importance of self‐caring processes in the construction of one's identity (Aguirre 2025; Gilbert 2010; Greenberg 2015; Timulak 2015). Self‐compassionate emotion states were linked with self‐knowledge and self‐awareness in clients with personality disorder (Kramer and Pascual‐Leone 2018). In a series of studies, client's access to self‐compassion has been related to good psychotherapy outcomes for this population (Kramer, Pascual‐Leone, Rohde, and Sachse 2016; Nardone et al. 2024), as well as more broadly across diagnoses (Han and Kim 2023). A meta‐analysis focusing on fostering self‐compassion in psychotherapy included *N* = 56 RCTs and showed medium effects on symptom reduction (i.e., reduction in anxiety and depression) at the end of the treatment (Han et al. 2023). It is interesting to note that the emergence of client's self‐compassion in the current study is specific to the treatment modality and may represent a feature of therapeutic attention given to the client's problems in GPM (see the effects for emergence of self‐compassion in GPM, Table 2B, whereas there is a reduction in the proportion of self‐compassion in TAU, Table 2B). This is consistent with the GPM model focusing on the BPD‐specific interpersonal hypersensitivity, which may contribute to the client feeling understood by the therapist, and they are, hence, indirectly fostering the emergence of self‐compassion and self‐understanding in the client, thus potentially strengthening their identity processes.

The emergence of clients' assertive anger in the current study is also consistent with the predictions of the sequential perspective of emotion processing in psychotherapy (Pascual‐Leone 2018, 2026). In an RCT on DBT, Kramer, Pascual‐Leone, Berthoud, et al. (2016) showed that the client's access to assertive anger contributed to the social rehabilitation in clients with BPD. Expressing one's healthy anger may seem counterintuitive when regarded as a ‘helpful’ stepping stone in the process of recovery, but is consistent with clinical wisdom. Linehan (1993) taught specific behavioural skills as part of the ‘DEAR MAN’ technique, which includes the expression of boundary‐setting emotions and behaviours (such as assertive anger and pride) as part of the interpersonal effectiveness module of DBT. It is noteworthy that the results of the present study suggest that the emergence of assertive anger is specific to the active treatment (and also to the fact of having a diagnosis) focusing on distinct emotion states within attachment relationships (i.e., the interpersonal hypersensitivity), which may contribute to (a) raised emotion awareness in the client, (b) specific awareness of what one needs in a conflictual interpersonal situation and (c) accessing and expressing boundary‐setting, identity‐supporting emotion states, such as assertive anger.

Emotion processing has been discussed as a mechanism of change from a transdiagnostic perspective (Sonderland et al. 2023); therapist fostering emotion processing was directly associated with distal therapy outcome (Iwakabe et al. 2023; Peluso and Freund 2018). For GPM specifically, helping the client to gain clarity over their activated emotional experiences, as they happen in the moment, is helpful and may contribute to interpersonal clarity and competence. This clarity is fostered by the GPM therapist when they focus on the client's interpersonal hypersensitivity within interpersonal relationships (Kramer, Grandjean, et al. 2024).

### 4.4 Clinical and Research Implications

The present research has several clinical and research implications. Firstly, it suggests that several psychotherapy models should focus more in‐depth on the process of undoing self‐contempt (Aguirre 2025), in particular for BPD (Wilner et al. 2024). Krawitz (2012) developed clinical recommendations for how to work with clients presenting with self‐contempt and self‐loathing. Although the present study was conducted in the context of GPM, its conclusions are valid for a large array of psychotherapy approaches, in particular for approaches that directly focus on emotion, such as DBT, compassion‐focused therapy and EFT.

Specifically for DBT (Linehan 1993), behaviour modification principles combined with Zen Buddhism are productive in addressing a wide range of emotional problems (Rudge et al. 2020), including the presence of self‐contempt in clients. Aguirre (2025) argues that this therapeutic focus on self‐contempt may be essential in certain clients, presenting with or without BPD. The present study may suggest that prosocial behaviour, trained in DBT skills using ‘GIVE’ and ‘DEAR MAN’ techniques, may be key to supporting individuals in making healthier and more effective interpersonal interactions, rather than falling back into their common self‐flagellation and severe self‐criticism, marked by contempt. The development of these novel skills in the client is key according to DBT.

Specifically for compassion‐focused psychotherapy (CFP; Gilbert 2010; Leaviss and Uttley 2015) for a broad array of psychological difficulties, the current study supports the idea of fostering self‐compassion as an effective antidote to self‐contemptuous states. Interestingly, for the treatment of severe complex trauma using an integrative version of DBT with elements of CFP, Bohus et al. (2020) propose fostering self‐compassion as an essential therapeutic strategy for facing the psychological difficulties in the aftermath of trauma. Again, these therapeutic strategies from CFP are conceived to enlarge the client's skills repertoire.

Specifically for EFT (Elliott et al. 2013; Greenberg 2015; Kramer and Timulak 2022; Pos and Greenberg 2012; Timulak 2015), focusing on particularly fragile and self‐destructive emotions as they emerge in the process of therapy is the experiential therapeutic groundwork from a transdiagnostic perspective, including personality disorders. With its radical focus on emotion, the treatment goal is less to teach the clients new skills on how to regulate certain intense emotions, but EFT works through processes of emotion sequences and memory reconsolidation related to past negative interpersonal experiences (Pascual‐Leone and Greenberg 2020), which may be particularly relevant for clients with personality disorders. As such, the focus on undoing self‐contempt may represent the peak of the iceberg of the full emotional experiences, where the client, step‐by‐step, accesses their emotional pain, their fear and shame, and then, their assertive anger and healthy grief. This process is assumed to take place in a prizing and valuing therapeutic relationship marked by therapeutic presence throughout, fostering the deep acceptance experientially and letting go of these painful experiences.

Finally, for research purposes, it is helpful to consider performance‐based assessment of emotion processing in the context of demonstrating a step‐by‐step change process in psychotherapy. Using an emotion‐evoking assessment procedure consistent with experiential theory (Kramer and Timulak 2024) will enable to study all emotion responses, as they arise, in the two‐chair dialogue. The present study has focused on a proposed sequence—undoing self‐contempt by assertive anger and self‐compassion—but the design allows researchers to explore other potential pathways of change that seem relevant. It also opens the way to study biological correlates of emotion changes in psychotherapy (e.g., Grandjean, Rohrbach, et al. 2025; Kim et al. 2020; Kramer, Renevey, and Pascual‐Leone 2020).

### 4.5 Limitations and Outlook

A number of limitations need to be acknowledged. All limitations discussed for the parent study apply to the present one. In particular, the small sample size prevented more advanced analyses explaining both the within‐person and the between‐person variance. Consistent with the design, we did not test the link with symptom outcome in the present study, but from the parent study, we know that advancement in emotion sequences mediated the reduction in interpersonal problems in GPM (Kramer, Grandjean, et al. 2024). Follow‐up analyses, including the usage of further treatment, at 1 year have not yet been included in the present study. The brief treatment context helped us to focus on a particularly relevant sequence, but for the study of maladaptive shame, it is probably needed to have longer psychotherapy processes, so that the impact of shame, and its changes, can emerge clearly. The following variables may have influenced the emotional experiences observed in the task during the present study: the client's intake capacity to regulate emotions (between‐person variance), the client's momentary quality of the therapeutic alliance with the therapist and the alliance with the assessor (within‐person variance). The formal assessment of on‐task investigator competence in conducting two‐chair dialogues has not been done in the present study, but clinical supervision ensured comparability of investigator interventions across participants. The limitation to two time points does not allow us to clearly disentangle the sequence between different types of emotion changes, but the design including the three groups allows for a cautious discussion of such a potential sequence. In future studies, three or more time points should be studied (see Haberman et al. 2019), applied to out‐of‐session assessments, to determine whether (a) self‐contempt changes first, then self‐compassion and assertive anger; (b) self‐compassion and assertive anger change first, which contribute to the decrease in self‐contempt; and (c) a third variable, for example, the increase and decrease over time of maladaptive shame represents the mediating link in this sequence, between the undoing of the self‐contempt and the emergence of the adaptive emotions.

To our knowledge, this is one of the first controlled studies to specifically investigate the undoing of a particularly elusive, yet essential and potentially harmful, component of emotion processing in psychotherapy: the client's self‐contempt assessed in the context of an experiential performance task. Despite limitations, repeated out‐of‐session assessments showed that undoing of self‐contempt happens to some degree in GPM for clients with BPD, and this process is supported by the emergence of self‐compassion and assertive anger. Although for healthy controls, the expressed self‐contempt and its reduction (which may be interpreted as a regression to the mean) do not have to be scaffolded by assertive anger, for the clients with BPD, the emergence of assertive anger and self‐compassion may be essential to undo the self‐contempt. GPM with its focus on the client's hypersensitivity and emotions may be one option among them. These results bear important clinical and research perspectives on targeting self‐contempt as a potentially destructive emotional state in BPD and other fragile presentations of the self, on the client's move towards healthier assertion and self‐compassion, supporting healthy identity processes.

## Funding

This study was supported by the Swiss National Science Foundation (SNSF100014_179457/1, to Pr. Kramer).

## Ethics Statement

The study was approved by the ethics committee CER‐VD (2017‐02167). Informed consent was given by all participants in writing to publish the results of the present study.

## Conflicts of Interest

The authors declare no conflicts of interest.

## Data Availability

The deidentified data stemming from this study are available upon request from the first author.
